# Back pain in seniors: the Back pain Outcomes using Longitudinal Data (BOLD) cohort baseline data

**DOI:** 10.1186/1471-2474-15-134

**Published:** 2014-04-23

**Authors:** Jeffrey G Jarvik, Bryan A Comstock, Patrick J Heagerty, Judith A Turner, Sean D Sullivan, Xu Shi, David R Nerenz, Srdjan S Nedeljkovic, Larry Kessler, Kathryn James, Janna L Friedly, Brian W Bresnahan, Zoya Bauer, Andrew L Avins, Richard A Deyo

**Affiliations:** 1 Department of Radiology, University of Washington, 325 Ninth Ave., Box 359728, Seattle, WA 98104-2499, USA; 2 Department of Neurological Surgery, University of Washington, Seattle, WA, USA; 3 Comparative Effectiveness, Cost and Outcomes Research Center, University of Washington, 325 Ninth Ave., Box 359728, Seattle, WA 98104-2499, USA; 4 Department of Biostatistics, University of Washington, Seattle, WA, USA; 5 Department of Psychiatry & Behavioral Sciences, University of Washington, Seattle, WA, USA; 6 Department of Rehabilitation Medicine, University of Washington, Seattle, WA, USA; 7 Department of Anesthesiology, Perioperative and Pain Medicine, Brigham and Women’s Hospital, Boston, MA, USA; 8 Neuroscience Institute, Henry Ford Hospital, Detroit, MI, USA; 9 Division of Research, Northern California Kaiser-Permanente, Oakland, CA, USA; 10 Department of Health Services, University of Washington, Seattle, WA, USA; 11 Department of Pharmacy, University of Washington, Seattle, WA, USA; 12 Departments of Family Medicine, Internal Medicine, and Public Health and Preventive Medicine, and the Center for Research in Occupational and Environmental Toxicology Oregon Health and Science University, Portland, OR, USA

## Abstract

**Background:**

Back pain represents a substantial burden globally, ranking first in a recent assessment among causes of years lived with disability. Though back pain is widely studied among working age adults, there are gaps with respect to basic descriptive epidemiology among seniors, especially in the United States. Our goal was to describe how pain, function and health-related quality of life vary by demographic and geographic factors among seniors presenting to primary care providers with new episodes of care for back pain.

**Methods:**

We examined baseline data from the Back pain Outcomes using Longitudinal Data (BOLD) registry, the largest inception cohort to date of seniors presenting to a primary care provider for back pain. The sample included 5,239 patients ≥ 65 years old with a new primary care visit for back pain at three integrated health systems (Northern California Kaiser-Permanente, Henry Ford Health System [Detroit], and Harvard Vanguard Medical Associates [Boston]). We examined differences in patient characteristics across healthcare sites and associations of patient sociodemographic and clinical characteristics with baseline patient-reported measures of pain, function, and health-related quality of life.

**Results:**

Patients differed across sites in demographic and other characteristics. The Detroit site had more African-American patients (50%) compared with the other sites (7-8%). The Boston site had more college graduates (68%) compared with Detroit (20%). Female sex, lower educational status, African-American race, and older age were associated with worse functional disability as measured by the Roland-Morris Disability Questionnaire. Except for age, these factors were also associated with worse pain.

**Conclusions:**

Baseline pain and functional impairment varied substantially with a number of factors in the BOLD cohort. Healthcare site was an important factor. After controlling for healthcare site, lower education, female sex, African-American race, and older age were associated with worse physical disability and all of these factors except age were associated with worse pain.

**Trial registration:**

Clinical Trials.gov NCT01776242; Registration date: June 13, 2012.

## Background

Back pain is the most common reason worldwide for years living with disability (YLDs) [1]. In the United States in 2010, low back pain was the top contributor to YLDs, outranking diseases such as chronic obstructive pulmonary disease and diabetes [2]. Although back pain is common among adults aged 65 years and older (“seniors”), it has been under-studied in this age group [3,4]. Recently, several publications from groups outside the U.S. have begun to explore this issue [5-7]. Nonetheless, relatively little is known concerning how back pain, function, and health-related quality of life (HRQoL) differ according to patient sociodemographic and clinical characteristics, especially among the elderly in the United States.

In this study, we addressed these knowledge gaps using baseline data from the Back pain Outcomes using Longitudinal Data (BOLD) cohort of patients aged 65 years and older initiating a new episode of care for back pain [8]. Our primary focus was to determine the associations of patient age, sex, race, ethnicity, and education with patient-reported measures of pain, function, and HRQoL. A unique strength of BOLD is the geographic diversity of the cohort participants. Therefore, we evaluated the magnitude of the differences in the characteristics of back pain patients across recruitment sites, the strength of the association between patient characteristics and baseline measures, and whether associations between patient characteristics and patient-reported outcomes are consistent across the three recruitment sites.

## Methods

### Institutional review board (IRB) approval

The study was approved by the Institutional Review Boards (IRBs) of all the participating institutions (University of Washington, Harvard Vanguard, Henry Ford Health Systems, Northern California Kaiser-Permanente).

### Patients and setting

We previously described the details of the registry [8]. In brief, using healthcare system electronic databases, we prospectively identified patients aged 65 years or older with a primary care visit for back pain in the prior 3 weeks at 3 integrated healthcare systems: Harvard Vanguard (Boston), Henry Ford Health Systems (Detroit), and Kaiser-Permanente Northern California. We excluded patients who had visits for back pain in the 6 months prior to the index visit. Our other primary inclusion and exclusion criteria were the International Classification of Diseases, Ninth Revision, Clinical Modification (ICD-9-CM) diagnostic codes [9] listed in Additional file 1. We approached patients in-person, by telephone or mail and performed and administered all baseline measures either in-person or by telephone within 3 weeks of their index visit, verifying eligibility and obtaining consent at that time. We enrolled patients from March 2011 through March 2013.

### Measures

We obtained the following patient-reported measures at baseline:

*Sociodemographic characteristics*: age, sex, race, ethnicity, education, employment status, marital status, smoking status and whether they had a lawyer involved with a back-related claim.

*Pain-related characteristics*: a) duration of current episode of back/leg pain (less than 1 month, 1–3 months, 3–6 months, 6–12 months, 1–5 years, more than 5 years); b) average back pain intensity and average leg pain intensity in the past week on 0–10 numerical rating scales where 0 = ‘no pain’ and 10 = ‘pain as bad as you can imagine’; c) the 24-item Roland-Morris Disability Questionnaire (RMDQ) [10], modified to specify disability related to either back or leg pain; and d) Brief Pain Inventory (BPI) Activity Interference Scale [11,12] (consisting of 7 0–10 ratings of how much pain interferes with general activity, mood, ability to walk, normal work, relations with other people, sleep and enjoyment of life).

*Psychological distress*: the 4-item PHQ-4 (0–12) measure of anxiety and depressive symptoms has been demonstrated to be a general marker of psychological distress [13]. PHQ-4 scores of 6 or greater have been recommended as “yellow flags” and scores of 9 or greater as “red flags” for presence of a depressive or anxiety disorder [14].

*HRQoL*: EuroQol-5D (EQ-5D), a preference-weighted, quality of life index (0–1) consisting of five dimensions (mobility, self-care, usual activities, pain/discomfort, and anxiety/depression) and a visual analog scale of current HRQoL [15].

*Falls*: Number of falls in the past 3 weeks and how many resulted in injury, from the Behavioral Risk Factor Surveillance System (BRFSS) survey [16].

*Recovery Expectations*: Patients rated their confidence that their back and/or leg pain would be completely gone or much better in 3 months on a scale from 0 = ‘not at all confident’ to 10 = ‘extremely confident’ [17,18].

### Statistical analysis

Due to the large sample size of the registry and known geographic and sociodemographic differences across the three recruitment sites, statistical comparisons of baseline variables across sites (ANOVA for continuous variables and Chi-square for categorical variables) yielded statistically significant differences at p = 0.05 on all measures except for age. We use boxplots (mean +/- standard deviation) and descriptive statistics to characterize RMDQ and back pain scores by patient sociodemographic characteristics and ICD-9-CM diagnostic codes assigned at the index visit. To examine the independent associations of baseline demographics with patient-reported outcome measures, we created separate multivariable linear regression models for RMDQ and back pain scores, adjusting for all available baseline demographic variables as well as recruitment site. We report unstandardized model coefficients and two-sided p-values without adjustment for multiple testing, because our research goal was to characterize the healthcare site differences rather than to test specific *a priori* hypotheses.

## Results

### Patient enrollment

**Figure 1:**
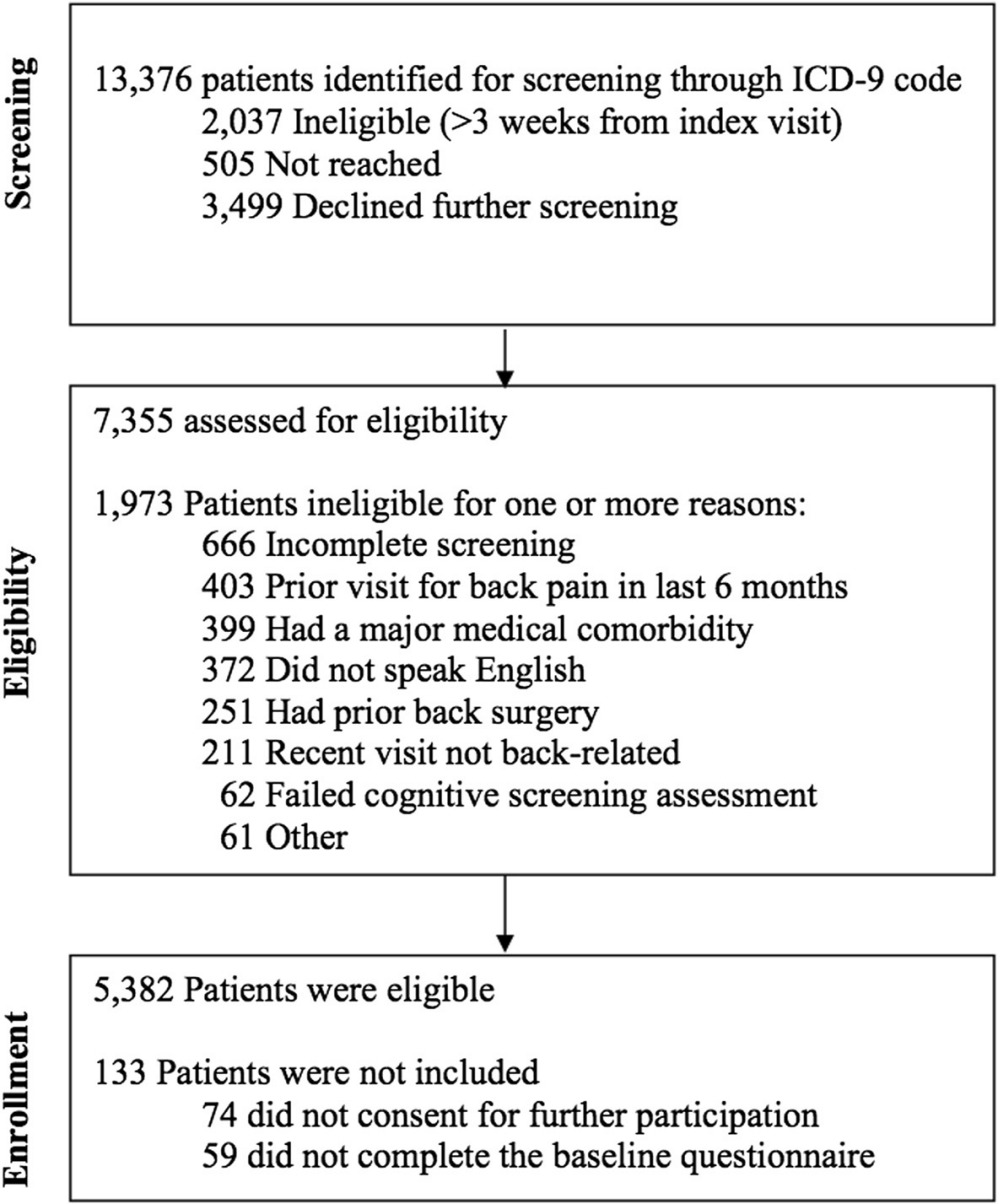
Consort diagram depicting patient flow from initial screening through enrollment.

Figure 1 depicts the flow of patients in the study. Of 13,376 patients identified as potentially eligible, we were unable to contact 15%, 15% were ineligible, and 27% declined to participate or to complete the baseline questionnaire. The remaining 5,239 patients (39% of patients identified for screening) enrolled and completed the baseline study measures. The mean (SD) number of days between the index visit and the baseline assessment was 14.6 (5.3).

### Patient differences across sites

Although patients from the three sites were similar in age, they differed on all other sociodemographic variables (Table 1). Notably, the Detroit site was characterized by the highest proportions of African-Americans (50%), females (70%), and current smokers (13%). The Boston site had more highly educated patients (68% with college degree), and more patients who were married (80%), followed in both cases by the Northern California site (39% with college degree, 58% married).

**Table 1:** BOLD cohort sociodemographic characteristics overall and by healthcare system

**Demographic variable**	**Combined**	**N. CA**	**Detroit**	**Boston**
	N = 5239*	**N = 3164**	**N = 967**	**N = 1108**
**Age, mean (SD) years**	73.8 ± 6.9	73.7 ± 6.8	73.8 ± 6.9	73.8 ± 7.1
**Sex, N (%)**				
**Male**	1852 (35%)	1154 (36%)	289 (30%)	409 (37%)
**Female**	3387 (65%)	2010 (64%)	678 (70%)	699 (63%)
**Hispanic, N (%)**	305 (6%)	270 (9%)	21 (2%)	14 (1%)
**Race, N (%)**				
**Black or African American**	797 (15%)	226 (7%)	485 (50%)	86 (8%)
**Native American Indian/Alaskan/Hawaiian or other Pacific Islander**	44 (1%)	40 (1%)	4 (0%)	0 (0%)
**Asian**	198 (4%)	171 (5%)	18 (2%)	9 (1%)
**Caucasian**	3843 (73%)	2430 (77%)	432 (45%)	981 (89%)
**Other**	299 (6%)	246 (8%)	26 (3%)	27 (2%)
**Education, N (%)**				
**Less than high school graduate**	314 (6%)	144 (5%)	154 (16%)	16 (1%)
**High school graduate or obtained a GED/Vocational, technical/trade school**	1450 (28%)	794 (25%)	382 (40%)	274 (25%)
**Some college**	1275 (24%)	978 (31%)	234 (24%)	63 (6%)
**Four year college graduate**	1290 (25%)	605 (19%)	89 (9%)	596 (54%)
**Professional or graduate degree**	894 (17%)	633 (20%)	105 (11%)	156 (14%)
**Marital, N (%)**				
**Married/living with a partner**	3191 (61%)	1850 (58%)	451 (47%)	890 (80%)
**Separated/divorced**	602 (11%)	423 (13%)	147 (15%)	32 (3%)
**Never married and presently single**	256 (5%)	163 (5%)	69 (7%)	24 (2%)
**Widowed**	1175 (22%)	716 (23%)	299 (31%)	160 (14%)
**Employment, N (%)**				
**Working full-time/part-time**	602 (11%)	367 (12%)	166 (15%)	69 (7%)
**Retired (not due to ill health)**	4250 (81%)	2535 (80%)	913 (82%)	802 (83%)
**Retired or disabled because of ill health**	146 (3%)	71 (2%)	12 (1%)	63 (7%)
**Other**	220 (4%)	177 (6%)	11 (1%)	32 (3%)
**Legal representation for back pain, N (%)**				
**Yes**	31 (1%)	23 (1%)	8 (1%)	0 (0%)
**No**	5196 (99%)	3136 (99%)	956 (99%)	1104 (100%)
**Smoking status, N (%)**				
**Never Smoked**	2901 (55%)	1570 (50%)	675 (70%)	656 (59%)
**Quit smoking over a year ago**	1999 (38%)	1422 (45%)	169 (17%)	408 (37%)
**Current smoker/quit less than a year ago**	324 (6%)	164 (5%)	121 (13%)	39 (4%)
**Days between index visit and baseline assessment, mean (SD)**	14.6 ± 5.3	16.0 ± 4.6	16.3 ± 5.3	8.9 ± 2.7

*Numbers vary slightly for items due to slight variation in response rates with overall missing items being less than 1%.

Detroit patients also differed from those at the other sites with respect to many of the patient-reported outcome measures (Table 2): they were less likely to have had back pain for less than 3 months, they rated their back and leg pain as more intense, they reported greater pain interference with activities, and they reported lower HRQoL, as measured by the EQ-5D. On the PHQ-4 measure of psychological distress [13,14], the mean score at the Boston site was lower than those at the other sites, but mean scores at each site were well below the “yellow flag” cutpoint of 6 points. The mean score on the recovery expectation rating was somewhat lower at the Detroit site than at the other sites.

**Table 2:** Patient-reported measures overall and by healthcare system site

**Baseline symptoms and outcomes**	**Combined**	**N. CA**	**Detroit**	**Boston**
	N = 5239*	**N = 3164**	**N = 967**	**N = 1108**
**RMDQ (0–24), mean (SD)**	9.5 ± 6.4	9.7 ± 6.0	12.8 ± 5.9	6.4 ± 6.4
**Back pain duration, N (%)**				
**< 1 month**	1749 (33%)	1215 (38%)	230 (24%)	304 (27%)
**1 - 3 months**	1015 (19%)	715 (23%)	137 (14%)	163 (15%)
**3 - 6 months**	344 (7%)	243 (8%)	46 (5%)	55 (5%)
**6 - 12 months**	313 (6%)	165 (5%)	96 (10%)	52 (5%)
**1 - 5 years**	776 (15%)	399 (13%)	197 (20%)	180 (16%)
**> 5 years**	1037 (20%)	426 (13%)	261 (27%)	350 (32%)
**Back pain intensity (0–10), mean (SD)**	5.0 ± 2.8	4.9 ± 2.7	6.2 ± 2.8	4.5 ± 2.7
**Leg pain intensity (0–10), mean (SD)**	3.4 ± 3.3	3.5 ± 3.2	4.2 ± 3.6	2.6 ± 3.1
**BPI Interference (0–10), mean (SD)**	3.3 ± 2.5	3.4 ± 2.5	3.8 ± 2.6	2.5 ± 2.3
**PHQ-4 (0–12), mean (SD)**	1.6 ± 2.5	1.9 ± 2.5	1.8 ± 2.8	0.4 ± 1.6
**EQ-5D index (-0.1-1), mean (SD)**	0.76 ± 0.17	0.76 ± 0.17	0.69 ± 0.20	0.81 ± 0.16
**EQ5D VAS (0–100), mean (SD)**	74.3 ± 18.4	73.6 ± 18.9	74.0 ± 18.4	76.6 ± 16.9
**Falls in past 3 weeks, N (%)**				
**Patients with one or more fall**	385 (7%)	288 (9%)	81 (8%)	16 (1%)
How many of these falls caused one or more injury**?	179 (46%)	121 (42%)	51 (63%)	7 (44%)
**Back Pain Recovery Expectations (0–10), mean (SD)**	5.5 ± 3.7	5.7 ± 3.6	4.9 ± 3.7	5.1 ± 4.0

• *Sample sizes vary slightly for items due to slight variation in response rates with overall missing items being less than 0.3%. Complete data were available for RMDQ, back pain intensity, and BPI interference.

• **The number and percent of patients in whom a fall caused at least one injury where injury was defined as limiting regular activities for at least a day or requiring a visit to a doctor.

### Relationship of patient sociodemographic characteristics to patient-reported pain, physical disability, and HRQoL

**Figure 2:**
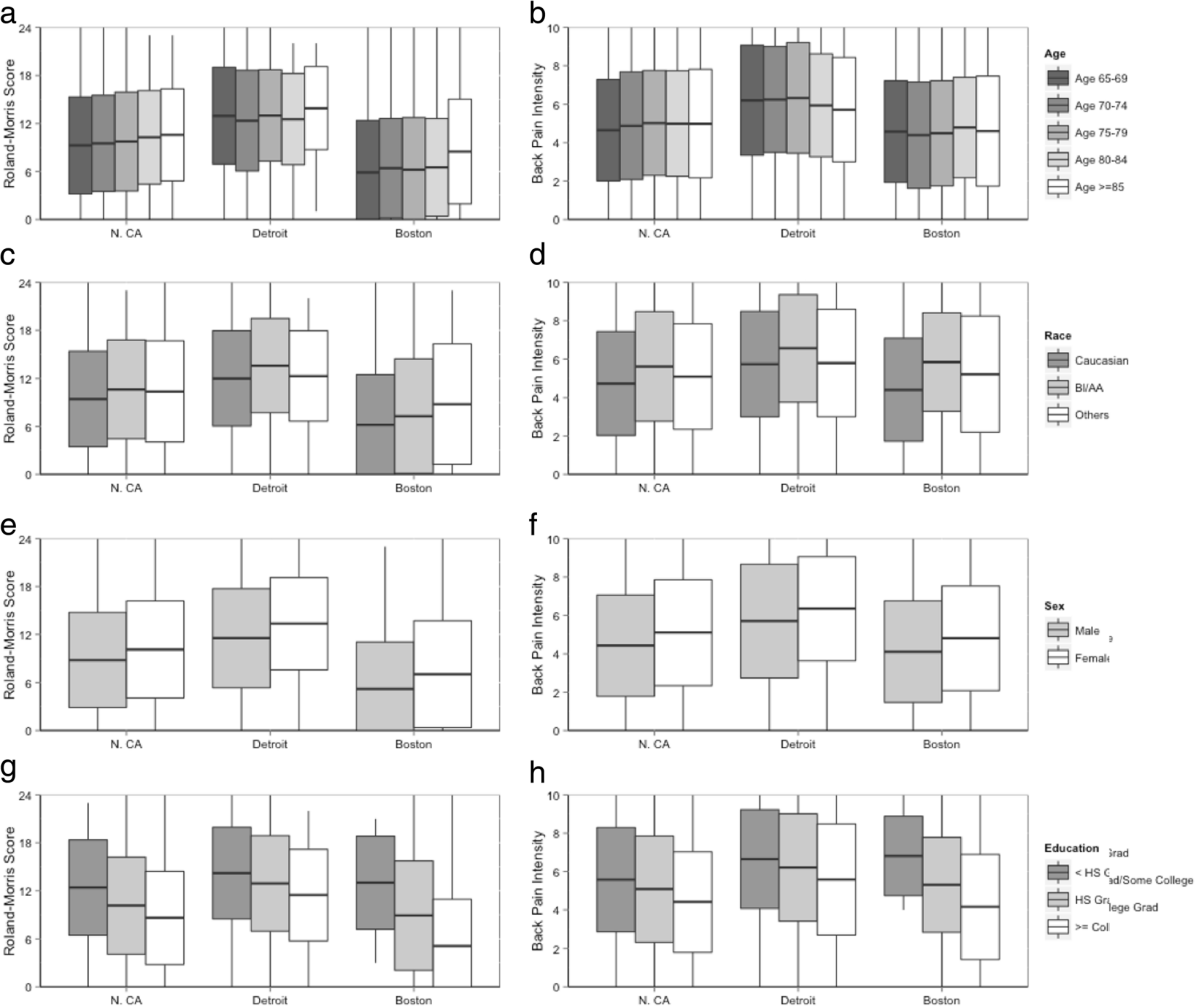
**Roland-Morris Disability Questionnaire (RMDQ) and average back pain intensity in past week by age, race, sex and education stratified by site. a**, **c**, **e**, and **g** display the baseline measure of back-related physical disability (the Roland-Morris disability questionnaire) and **b**, **d**,** f**, **h** display the baseline measure of pain (a numerical rating scale of average back pain in past week), stratified by site and compared with key demographic variables. **a** and **b** depict baseline measures by age; **c** and **d** depict baseline measures by race; **e** and **f** depict baseline measures by sex; and **g** and **h** depict baseline measures by education. Each outcome by demographic boxplot displays the within-group mean (horizontal in the center of the box) +/- the within-group standard deviation (upper and lower edges of the box). The vertical lines display the within-group range of scores.

Figure 2 shows RMDQ and back pain intensity scores at each site stratified by patient age, race, sex, and education. RMDQ scores increased with age, from a mean (SD) of 9.2 (6.6) among those aged 65–69 to 10.7 (6.1) for those older than 85 across sites. The oldest age group (≥85) had worse RMDQ scores at all three sites. The average pain duration also increased with age, with 31% of patients in the youngest age group (65–69) having had pain more than one year versus 44% in the oldest age group (≥85). The EQ-5D index was similar for all age groups [mean (SD) = 0.76 (0.17)].

African-Americans reported worse pain and pain-related physical disability than did Caucasians at all study sites (Figure 2). The mean (SD) RMDQ score was 12.1 (6.5) among African-Americans compared with 8.9 (6.3) for Caucasians. The proportion of African-American patients whose pain duration was longer than a year was 42% compared with 34% for Caucasians, and the mean scores for back and leg pain were 0.9 and 1.0 points worse, respectively, for African-Americans than for Caucasians after adjusting for site.

We observed these relationships at each site as well as for the overall cohort. For example, although African-Americans at the Detroit site reported greater physical disability than did African-Americans at the Northern California site (RMDQ mean (SD) = 13.6 (5.9) versus10.6 (6.2)) or Boston (7.3 (7.1)), within each site, RMDQ scores were higher for African-Americans than for Caucasians (Detroit 13.6 (5.9) vs. 12.0 (5.9), Northern California 10.6 (6.2) vs. 9.4 (6.0), and Boston 7.3 (7.1) vs. 6.2 (6.3)).

Patients from different racial groups differed in education. More Caucasians (47%) than African-Americans (19%) were college graduates.

Because of the small number of Hispanic patients at Detroit (21) and Boston (14), we report data from Hispanics from Northern California only. The mean RMDQ score was 1.2 points higher (worse) for Hispanics than for non-Hispanic Caucasians [10.7 (6.0) vs. 9.5 (6.0)]. Hispanics also reported greater back pain intensity, activity interference, and depression/anxiety.

Women reported significantly worse function, pain, and activity interference (Figure 2e and f). However, men and women did not differ in back pain duration, PHQ-4 scores, EQ5D scores, or recovery expectations.

Although there were marked differences across sites in levels of education (Figure 2g and h), both across and within sites, patients with less than high school education reported the greatest pain duration, back pain, leg pain, and physical disability. Among patients with less than high school education, 51% reported pain duration greater than 1 year, compared with only 27% among those with a college degree.

Patients who had retired or were disabled due to ill health had worse pain and physical function than patients still working. Patients who had retired not due to ill health also had somewhat worse pain and physical function than did patients still working.

### Association of diagnosis with pain, function, and HRQoL

**Figure 3:**
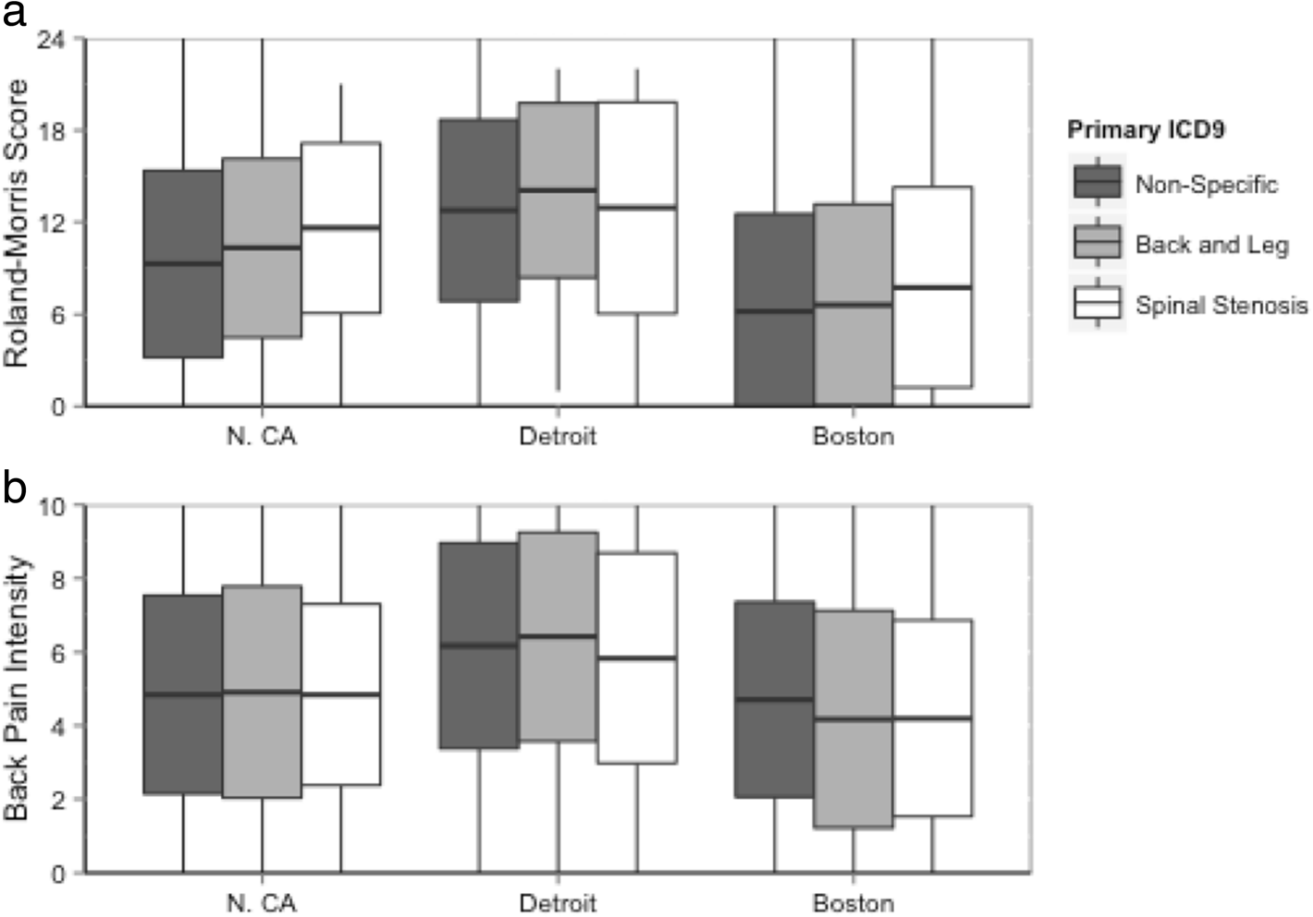
**RMDQ and back pain intensity by ICD-9-CM code stratified by site. a** displays the baseline measure of back-related physical disability (the Roland-Morris disability questionnaire) and **b** displays the baseline measure of pain (a numerical rating scale of average back pain in past week), stratified by site and compared with primary diagnosis code for the index visit using ICD-9-CM. Each outcome by demographic boxplot displays the within-group mean (horizontal in the center of the box) +/- the within-group standard deviation (upper and lower edges of the box). The vertical lines display the within-group range of scores.

Table 3 lists the six most commonly recorded diagnosis codes (see Additional file 1 for a complete list of ICD-9-CM Diagnosis Codes). Lumbago was used more than three times as frequently as the next most commonly used code. Patients with the diagnosis codes that indicated leg involvement (sciatica, back pain with radiation, and stenosis) had higher leg pain intensity ratings than did patients with diagnoses that did not include leg involvement (lumbago, backache, strain and sprain). Patients with diagnosis codes indicating back pain with leg involvement as well as stenosis diagnoses codes had the highest RMDQ scores (mean (SD) = 10.0 (6.4) and 10.0 (6.2), respectively) (Figure 3).

**Table 3:** ICD-9-CM Diagnosis codes

**Diagnosis codes**	**N (%)**	**RMDQ**	**Back pain intensity**	**Leg pain intensity**
**Non-Specific: lumbago, backache, sprains and strains**	3560 (67.95%)	4.0 9.0 15.0 (9.5 ± 6.5)*	3.0 5.0 7.0 (5.1 ± 2.8)	0.0 2.0 6.0 (3.0 ± 3.3)
**Back and Leg: back pain with radiation, sciatica**	1091 (20.82%)	5.0 10.0 15.0 (10.0 ± 6.2)	3.0 5.0 7.0 (4.9 ± 2.9)	3.0 5.0 7.0 (5.1 ± 3.0)
**Spinal Stenosis**	288 (5.50%)	5.0 10.0 15.2 (10.0 ± 6.4)	3.0 5.0 6.0 (4.6 ± 2.6)	0.0 5.0 6.0 (4.0 ± 3.2)
**Others**	300 (5.73%)	2.0 7.0 13.0 (8.0 ± 6.6)	3.0 5.0 7.0 (4.7 ± 2.7)	0.0 0.0 4.0 (2.2 ± 2.9)

*25^th^ percentile, median, 75^th^ percentile (mean ± standard deviation).

### Multivariable analysis of association of baseline characteristics with RMDQ and pain

The multivariable analysis demonstrated strong associations of site, employment status, education and duration of symptoms with RMDQ scores after adjustment for other baseline demographic variables (Table 4). We also observed modest associations of race, age, gender and smoking status with RMDQ scores. We observed similar patterns of association for back pain intensity ratings, although, unlike for RMDQ scores, back pain intensity was negatively associated with the number of days since the index visit and was not associated with age. Neither marital status nor Hispanic ethnicity was associated with RMDQ scores or back pain intensity.

**Table 4:** Multivariable linear regression for dependent variables of baseline RMDQ and back pain NRS on patient characteristics

**Independent variable**	**Levels**	**Dependent variable: RMDQ (0–24)**		**Dependent variable: back pain intensity (0–10)**	
		Coef* (95% CI)	**P-value**	Coef* (95% CI)	**P-value**
**Site**	**Boston**	Ref	<0.001	Ref	<0.001
	**Detroit**	5.22 (4.56, 5.88)		1.26 (0.96, 1.56)	
	**N. CA**	3.30 (2.77, 3.82)		0.59 (0.35, 0.83)	
**Age**	***(Per year)***	0.03 (0.00, 0.05)	0.048	0.00 (-0.01, 0.01)	0.983
**Sex**	**Male**	Ref	<0.001	Ref	<0.001
	**Female**	1.10 (0.73, 1.46)		0.50 (0.34, 0.67)	
**Hispanic**	**Yes**	Ref	0.578	Ref	0.087
	**No**	-0.24 (-1.10, 0.62)		-0.34 (-0.73, 0.05)	
**Race**	**Black or African American**	Ref	0.011	Ref	<0.001
	**Native American Indian/Alaskan/Hawaiian or other Pacific Islander**	-0.13 (-1.93, 1.67)		-0.05 (-0.88, 0.77)	
	**Asian**	-0.29 (-1.25, 0.68)		-0.50 (-0.94, -0.06)	
	**Caucasian**	-0.76 (-1.27, -0.25)		-0.72 (-0.96, -0.49)	
	**Other**	0.16 (-0.79, 1.10)		-0.51 (-0.94, -0.07)	
**Education**	**Less than high school graduate**	Ref	<0.001	Ref	<0.001
	**High school graduate/obtained a GED/Vocational, technical, or trade school**	-1.13 (-1.87, -0.39)		-0.35 (-0.69, -0.02)	
	**Some college**	-2.20 (-2.96, -1.44)		-0.40 (-0.75, -0.06)	
	**Four year college graduate**	-2.99 (-3.78, -2.20)		-0.80 (-1.16, -0.44)	
	**Professional or graduate degree**	-3.04 (-3.85, -2.23)		-1.05 (-1.42, -0.68)	
**Marital**	**Married/living with a partner**	Ref	0.386	Ref	0.086
	**Separated/divorced**	0.20 (-0.34, 0.73)		0.07 (-0.17, 0.32)	
	**Never married and presently single**	0.45 (-0.31, 1.21)		0.25 (-0.10, 0.60)	
	**Widowed**	0.33 (-0.12, 0.78)		0.25 (0.04, 0.45)	
**Employment**	**Working full-time/part-time**	Ref	<0.001	Ref	0.003
	**Retired (not due to ill health)**	0.86 (0.33, 1.38)		0.30 (0.06, 0.54)	
	**Retired or disabled because of ill health**	3.90 (2.82, 4.99)		0.87 (0.38, 1.37)	
	**Other**	0.60 (-0.33, 1.54)		0.08 (-0.34, 0.51)	
**Legal rep.**	**Yes**	Ref	0.059	Ref	0.115
	**No**	-2.00 (-4.07, 0.08)		-0.76 (-1.71, 0.19)	
**Smoking status**	**Never Smoked**	Ref	<0.001	Ref	0.012
	**Quit smoking over a year ago**	0.39 (0.04, 0.74)		0.13 (-0.03, 0.29)	
	**Current smoker/quit less than a year ago**	1.46 (0.77, 2.15)		0.45 (0.14, 0.77)	
**ICD9 code**	**Non-specific**	Ref	<0.001	Ref	0.051
	**Back and Leg**	1.00 (0.59, 1.42)		-0.04 (-0.23, 0.15)	
	**Spinal Stenosis**	1.08 (0.35, 1.81)		-0.46 (-0.79, -0.13)	
	**Others**	-0.75 (-1.46, -0.05)		-0.14 (-0.47, 0.18)	
**Pain duration**	**0 - 1 month**	Ref	<0.001	Ref	<0.001
	**1 - 3 month**	0.62 (0.16, 1.08)		0.13 (-0.08, 0.34)	
	**3 - 6 month**	0.61 (-0.07, 1.30)		0.33 (0.02, 0.65)	
	**6 - 12 month**	1.07 (0.35, 1.78)		0.42 (0.10, 0.75)	
	**1 - 5 years**	1.80 (1.29, 2.31)		0.46 (0.23, 0.69)	
	**> 5 years**	2.03 (1.56, 2.50)		0.53 (0.31, 0.74)	
**Time from index visit to assessment** ***(per day, 0–21)***		-0.04 (-0.08, -0.00)	0.029	-0.07 (-0.08, -0.05)	<0.001

*Multivariable regression model coefficients are on the scale of the dependent variable and are not standardized.

## Discussion

This is the largest cohort to date of adults aged 65 and older with a new episode of care for back pain. Our results show that there are important differences in pain intensity, physical disability, and health-related quality of life across different healthcare sites and across different patient age, sex, and racial subgroups. These findings are of importance to both researchers and clinicians. For researchers comparing the effectiveness of interventions in observational studies, our findings emphasize the importance of adjusting for any patient baseline differences between treatment groups in factors that are associated with outcome measures of pain, function, and HRQoL. For example, education level appears to be important in this regard, but often is not reported or adjusted for in such studies. Our findings also indicate that identifiable subgroups of older patients may differ substantially at the time of initiating a new episode of care for back pain. This suggests the potential value of applying different interventions tailored to these different subgroups. Moreover, the baseline characteristics of patients and the healthcare system environment in which they are located should be considered when evaluating treatment outcomes.

We observed sizable differences in baseline patient-reported measures across recruitment sites, with patients from the Detroit site worse on most measures as compared to the Boston and Northern California sites. Site differences in disability and pain persisted even after controlling for demographic factors available to us. It is possible that unmeasured socioeconomic differences are responsible at least in part for the site differences. Detroit was suffering from a severe economic recession during the study time-frame, and depressed economic conditions have been shown to be associated with poorer well-being and quality of life in the elderly [19]. Our findings emphasize the limitations of single-site observational studies, which may not be generalizable to other settings.

Our finding that less educated patients reported worse function is concordant with a review by Dionne and colleagues of education and back pain [20]. They speculated that education may be a marker for other factors, such as ability to adapt to stress, access to healthcare, occupational factors, and behavioral/environmental factors. All patients in our study had access to healthcare. However, it is possible that less educated patients also were financially disadvantaged and may have delayed care due to concerns about having to pay co-insurance costs. Less educated patients may be economically disadvantaged and under more psychosocial stress, which could affect health outcomes. Another possibility is that the association of lower education with worse health-related measures reflects the cumulative effects of social disadvantage on disease burden [21].

The finding that women present with worse pain and physical disability than men is consistent with prior research in largely younger populations [22-24].

While there was increasing pain-related physical disability (RMDQ score) with age, pain severity was not clearly associated with age. Prior studies in conditions other than back pain have yielded conflicting findings regarding the association between age and pain severity. Creamer found no association between age and pain severity in a sample of patients with knee osteoarthritis [25]. Thomas [26] and Parsons [27] found increasing pain severity with increasing age for a variety of musculoskeletal conditions. In a nationally representative sample of Medicare beneficiaries, pain reporting did not vary by age [28].

Somewhat surprising was that, independent of age, patients who had retired for reasons other than ill health also had worse pain and physical disability than those patients still working. While this might be due in part to the healthy worker effect [29], other authors have found retirement associated with a variety of symptoms such as declining mobility and daily activities, and declining mental health [30], including depression [31].

In our multivariable analysis, we found that even after adjusting for site, education, pain duration and other factors, African-American race was associated with worse baseline physical disability and pain at presentation for back pain-related care. This finding is consistent with other studies that found worse pain-related disability in cohorts of African-Americans compared with other races [32-35]. Thus, our observation of worse pain and physical disability among African-Americans compared with Caucasians could be explained by different coping strategies, or could be a result of residual confounding in our multivariable analysis.

Patients with diagnosis codes indicating leg involvement or spinal stenosis reported slightly worse physical disability. This is concordant with other studies indicating that patients with leg involvement have more severe pain and physical disability than those without [36-38].

Adjusting for other variables, as compared with patients who never smoked, those who were former smokers reported somewhat higher levels of physical disability and pain, and current smokers reported even higher levels of disability and pain. Substantial prior research has linked smoking to worse back pain outcomes, and one study of patients with spine-related back or leg pain found that compared with patients who had never smoked, current smokers reported greater pain; in longitudinal analyses, compared with patients who continued to smoke, those who quit reported significantly greater improvement in pain [39].

It is worth emphasizing that our large sample size allowed us to detect what were frequently relatively small differences in patient reported measures between subgroups. The importance of the magnitude of these differences on an individual level is uncertain.

One of the limitations of our project is that we enrolled nearly two-thirds of the cohort from a single site – Northern California Kaiser-Permanente. A second limitation is that nearly all of the Hispanics were from one site (Northern California) and a majority of African-Americans were from another site (Detroit). This distribution of patients limits the overall generalizability of our findings. Differences observed in self-reported measures and outcomes may reflect site-specific differences that are based on local healthcare system or patient-specific factors. Because patients at the 3 sites had different sociodemographic characteristics, we will need to control for these factors in future analyses. A third limitation is that we enrolled 39% of patients initially identified as potentially eligible. Because these patients did not complete questionnaires, we cannot further characterize this non-enrolled group, but we acknowledge that they potentially limit generalizability.

Another limitation is that this study was not designed to determine sociodemographic differences between subgroups with low back pain, but rather was designed to examine the natural history of back pain among seniors at three integrated health systems. Subgroup differences may reflect institutional and other local factors that we did not measure, such as income and co-pays, that could influence access to healthcare and hence utilization and outcomes.

While ours is the first cohort of seniors with back pain assembled from a primary care setting in the United States, there have been similar cohorts assembled internationally. The Back Complaints in the Elders (BACE) [40] group is a consortium of investigators from the Netherlands, Australia and Brazil who are assembling similar but smaller cohorts, planned to be around 750 patients per national cohort. The first of these to be published was the Dutch BACE cohort that enrolled 675 patients [7]. Their inclusion criteria were similar to ours, recruiting primary care patients with a new episode of care for back pain. They included slightly younger patients (>55 years old). They also had a slightly shorter allowed interval between the index visit and when they contacted patients, allowing a maximum of 2 weeks compared with 3 weeks for our study. These studies will provide an opportunity to compare and contrast the presentation, diagnosis and treatments of seniors with back pain between the U.S. and other countries.

## Conclusions

In summary, we have presented the BOLD baseline results describing the demographic and baseline patient-reported measures. Site was strongly associated with baseline patient reported measures. It is unclear whether site differences were due to geographical factors, socio-cultural factors specific to a particular area, or factors specific to the healthcare system that oversees the care of these patients. Adjusting for site reveals continued association among key demographic variables of race, age, and education with presenting pain. Our results lay the groundwork for future studies involving this cohort.

## Abbreviations

ANOVA: Analysis of variance; BACE: Back complaints in the elders; BOLD: Back pain Outcomes using Longitudinal Data; BPI: Brief pain inventory; BRFSS: Behavioral Risk Factor Surveillance System; HRQoL: Health-related quality of life; ICD-9-CM: International classification of diseases, ninth revision, clinical modification; IRB: Institutional review board; NRS: Numerical rating scale; PHQ-4: Patient health questionnaire for depression and anxiety- 4 item; RMDQ: Roland-Morris Disability Questionnaire; SD: Standard deviation; YLD: Years living with disability.

## Competing interests

The authors declare that they have no competing interests.

Dr. Jarvik has the following potential conflicts of interest, although they do not relate directly to the subject of this manuscript, he lists them in the spirit of full disclosure. He served on the Comparative Effectiveness Advisory Board for GE Healthcare through October 2012. He is a co-founder and stockholder of PhysioSonics, a high intensity focused ultrasound company, and receives royalties for intellectual property. He is also a consultant for HealthHelp, a radiology benefits management company.

## Authors’ contributions

JGJ, BAC, PJH, JAT, SDS, DRN, SSN, LK, JLF, BWB, ALA, and RAD developed the original concept of the study and developed the design of BOLD Registry study. ZB and KJ participated in the design of as BOLD and are project directors. XS participated in the data analysis and report writing. All authors have read and approved the final version of the article.

## Pre-publication history

The pre-publication history for this paper can be accessed here:

http://www.biomedcentral.com/1471-2474/15/134/prepub

## Supplementary Material

Additional file 1 **Baseline diagnostic categories and ICD-9 CM diagnosis codes included in each.** The diagnoses in italics accounted for approximately 80% of subjects. Click here for file

## References

[B1] Vos T Flaxman AD Naghavi M Lozano R Michaud C Ezzati M Shibuya K Salomon J Abdalla S Aboyans V Abraham J Ackerman I Aggarwal R Ahn S Ali M AlMazroa M Alvarado M Anderson H Anderson L Andrews K Atkinson C Baddour L Bahalim A Barker-Collo S Barrero L Bartels D Basáñez M-G Baxter A Bell M Benjamin E Years lived with disability (YLDs) for 1160 sequelae of 289 diseases and injuries 1990–2010: a systematic analysis for the global burden of disease study 2010 Lancet 2012 380 2163 2196 23245607 10.1016/S0140-6736(12)61729-2 PMC6350784

[B2] Collaborators USBoD The state of US health, 1990–2010: burden of diseases, injuries, and risk factors JAMA 2013 310 591 608 23842577 10.1001/jama.2013.13805 PMC5436627

[B3] Bressler HB Keyes WJ Rochon PA Badley E The prevalence of low back pain in the elderly: a systematic review of the literature Spine (Phila Pa 1976) 1999 24 1813 1819 10488512 10.1097/00007632-199909010-00011

[B4] Scheele J Luijsterburg PA Bierma-Zeinstra SM Koes BW Course of back complaints in older adults: a systematic literature review Eur J Phys Rehabil Med 2012 48 379 386 22820821

[B5] Macfarlane GJ Beasley M Jones EA Prescott GJ Docking R Keeley P McBeth J Jones G The prevalence and management of low back pain across adulthood: results from a population-based cross-sectional study (the MUSICIAN study) Pain 2012 153 27 32 21978663 10.1016/j.pain.2011.08.005

[B6] Scheele J Enthoven WT Bierma-Zeinstra SM Peul WC van Tulder MW Bohnen AM Berger MY Koes BW Luijsterburg PA Course and prognosis of older back pain patients in general practice: A prospective cohort study Pain 2013 154 6 951 957 23597679 10.1016/j.pain.2013.03.007

[B7] Scheele J Enthoven WT Bierma-Zeinstra SM Peul WC van Tulder MW Bohnen AM Berger MY Koes BW Luijsterburg PA Characteristics of older patients with back pain in general practice: BACE cohort study Eur J Pain 2014 18 2 279 287 23868792 10.1002/j.1532-2149.2013.00363.x

[B8] Jarvik JG Comstock BA Bresnahan BW Nedeljkovic SS Nerenz DR Bauer Z Avins AL James K Turner JA Heagerty P Kessler L Friedly JL Sullivan SD Deyo RA Study protocol: the back pain outcomes using longitudinal data (BOLD) registry BMC Musculoskelet Disord 2012 13 64 22554166 10.1186/1471-2474-13-64 PMC3403933

[B9] Statistics NCfH International classification of diseases, ninth revision (ICD-9)], 2009 Available at: http://www.cdc.gov/nchs/icd/icd9.htm 10.7326/0003-4819-88-3-424 629506

[B10] Roland M Morris R A study of the natural history of back pain: part 1: development of a reliable and sensitive measure of disability in low back pain Spine 1983 8 141 144 6222486 10.1097/00007632-198303000-00004

[B11] Cleeland CS Nakamura Y Mendoza TR Edwards KR Douglas J Serlin RC Dimensions of the impact of cancer pain in a four country sample: new information from multidimensional scaling Pain 1996 67 267 273 8951920 10.1016/0304-3959(96)03131-4

[B12] Cleeland CS Ryan KM Pain assessment: global use of the brief pain inventory Ann Acad Med Singapore 1994 23 129 138 8080219

[B13] Kroenke K Spitzer RL Williams JB Lowe B An ultra-brief screening scale for anxiety and depression: the PHQ-4 Psychosomatics 2009 50 613 621 19996233 10.1176/appi.psy.50.6.613

[B14] Lowe B Wahl I Rose M Spitzer RL Glaesmer H Wingenfeld K Schneider A Brahler E A 4-item measure of depression and anxiety: validation and standardization of the patient health questionnaire-4 (PHQ-4) in the general population J Affect Disord 2010 122 86 95 19616305 10.1016/j.jad.2009.06.019

[B15] Brooks R EuroQOL: the current state of play Health Policy 1996 37 53 72 10158943 10.1016/0168-8510(96)00822-6

[B16] Stevens JA Mack KA Paulozzi LJ Ballesteros MF Self-reported falls and fall-related injuries among persons aged > or =65 years--United States, 2006 CDC MMWR Morb Mortal Wkly Rep 2008 57 225 229 18322444

[B17] Iles RA Davidson M Taylor NF O’Halloran P Systematic review of the ability of recovery expectations to predict outcomes in non-chronic non-specific low back pain J Occup Rehabil 2009 19 25 40 19127345 10.1007/s10926-008-9161-0

[B18] Kongsted A Vach W Axo M Bech RN Hestbaek L Expectation of recovery from low back pain: a longitudinal cohort study investigating patient characteristics related to expectations and the association between expectations and 3-month outcome Spine (Phila Pa 1976) 2014 39 81 90 24108283 10.1097/BRS.0000000000000059

[B19] Fenge LA Hean S Worswick L Wilkinson C Fearnley S Ersser S The impact of the economic recession on well-being and quality of life of older people Health Soc Care Community 2012 20 617 624 22891952 10.1111/j.1365-2524.2012.01077.x

[B20] Dionne CE Von Korff M Koepsell TD Deyo RA Barlow WE Checkoway H Formal education and back pain: a review J Epidemiol Community Health 2001 55 455 468 11413174 10.1136/jech.55.7.455 PMC1731944

[B21] Lacey RJ Belcher J Croft PR Does life course socio-economic position influence chronic disabling pain in older adults? A general population study Eur J Pub Health 2013 23 534 540 22874735 10.1093/eurpub/cks056 PMC3719471

[B22] Bartley EJ Fillingim RB Sex differences in pain: a brief review of clinical and experimental findings Br J Anaesth 2013 111 52 58 23794645 10.1093/bja/aet127 PMC3690315

[B23] Fillingim RB King CD Ribeiro-Dasilva MC Rahim-Williams B Riley JL Sex, gender, and pain: a review of recent clinical and experimental findings J Pain 2009 10 447 485 19411059 10.1016/j.jpain.2008.12.001 PMC2677686

[B24] Hurley RW Adams MC Sex, gender, and pain: an overview of a complex field Anesth Analg 2008 107 309 317 18635502 10.1213/01.ane.0b013e31816ba437 PMC2715547

[B25] Creamer P Lethbridge-Cejku M Hochberg MC Determinants of pain severity in knee osteoarthritis: effect of demographic and psychosocial variables using 3 pain measures J Rheumatol 1999 26 1785 1792 10451078

[B26] Thomas E Peat G Harris L Wilkie R Croft PR The prevalence of pain and pain interference in a general population of older adults: cross-sectional findings from the North Staffordshire osteoarthritis project (NorStOP) Pain 2004 110 361 368 15275787 10.1016/j.pain.2004.04.017

[B27] Parsons S Breen A Foster NE Letley L Pincus T Vogel S Underwood M Prevalence and comparative troublesomeness by age of musculoskeletal pain in different body locations Fam Pract 2007 24 308 316 17602173 10.1093/fampra/cmm027

[B28] Patel KV Guralnik JM Dansie EJ Turk DC Prevalence and impact of pain among older adults in the United States: Findings from the 2011 National Health and Aging Trends Study Pain 2013 154 12 2649 2657 24287107 10.1016/j.pain.2013.07.029 PMC3843850

[B29] Li CY Sung FC A review of the healthy worker effect in occupational epidemiology Occup Med 1999 49 225 229 10.1093/occmed/49.4.225 10474913

[B30] Dave D Rashad I Spasojevic J The effects of retirement on physical and mental health outcomes NBER working paper series 2006 Cambridge, MA: National Bureau of Economic Research

[B31] Christ SL Lee DJ Fleming LE LeBlanc WG Arheart KL Chung-Bridges K Caban AJ McCollister KE Employment and occupation effects on depressive symptoms in older Americans: does working past age 65 protect against depression? J Gerontol B Psychol Sci Soc Sci 2007 62 S399 S403 18079428 10.1093/geronb/62.6.s399

[B32] Olsen TL Anderson RL Dearwater SR Kriska AM Cauley JA Aaron DJ LaPorte RE The epidemiology of low back pain in an adolescent population Am J Public Health 1992 82 606 608 1532116 10.2105/ajph.82.4.606 PMC1694113

[B33] Baker TA Green CR Intrarace differences among black and white americans presenting for chronic pain management: the influence of age, physical health, and psychosocial factors Pain Med 2005 6 29 38 15669948 10.1111/j.1526-4637.2005.05014.x

[B34] Waterman BR Belmont PJ Jr Schoenfeld AJ Low back pain in the United States: incidence and risk factors for presentation in the emergency setting Spine J 2012 12 63 70 21978519 10.1016/j.spinee.2011.09.002

[B35] Carey TS Freburger JK Holmes GM Jackman A Knauer S Wallace A Darter J Race, care seeking, and utilization for chronic back and neck pain: population perspectives J Pain 2010 11 343 350 19853527 10.1016/j.jpain.2009.08.003 PMC2847652

[B36] Grotle M Brox JI Veierod MB Glomsrod B Lonn JH Vollestad NK Clinical course and prognostic factors in acute low back pain: patients consulting primary care for the first time Spine (Phila Pa 1976) 2005 30 976 982 15834343 10.1097/01.brs.0000158972.34102.6f

[B37] Selim AJ Fincke G Ren XS Deyi RA Lee A Skinner K Kazis L Patient characteristics and patterns of use for lumbar spine radiographs: results from the veterans health study Spine 2000 25 2440 2444 11013494 10.1097/00007632-200010010-00004

[B38] Tubach F Beaute J Leclerc A Natural history and prognostic indicators of sciatica J Clin Epidemiol 2004 57 174 179 15125627 10.1016/S0895-4356(03)00257-9

[B39] Behrend C Prasarn M Coyne E Horodyski M Wright J Rechtine GR Smoking cessation related to improved patient-reported pain scores following spinal care J Bone Joint Surg Am 2012 94 2161 2166 23095839 10.2106/JBJS.K.01598

[B40] Scheele J Luijsterburg PA Ferreira ML Maher CG Pereira L Peul WC van Tulder MW Bohnen AM Berger MY Luijsterburg PA Koes BW Back complaints in the elders (BACE); design of cohort studies in primary care: an international consortium BMC Musculoskelet Disord 2011 12 193 21854620 10.1186/1471-2474-12-193 PMC3182961

