# Supplementary material for: Back pain in seniors: the Back pain Outcomes using Longitudinal Data (BOLD) cohort baseline data
Source: BMC Musculoskelet Disord. 2014 Apr 23;15:134. doi: 10.1186/1471-2474-15-134 (PMC4021204; doi:10.1186/1471-2474-15-134)
Supplement: Additional file 1 — Baseline diagnostic categories and ICD-9 CM diagnosis codes included in each. The diagnoses in italics accounted for approximately 80% of subjects. [file 1471-2474-15-134-S1.docx]

**Appendix**: Baseline diagnostic categories and ICD-9 CM diagnosis codes included in each. The diagnoses in italics accounted for approximately 80% of subjects.

| **Back Pain alone** | |  | **Lumbar stenosis** | | |
| --- | --- | --- | --- | --- | --- |
| 307.89 | Pain disorders related to psychological factors, other |  | 724.0 | | Spinal stenosis other than cervical |
| 721.3 | Lumbosacral spondylosis without myelopathy |  | 724.00 | | Spinal stenosis, unspecified region |
|  |  |  | 724.01 | | Thoracic stenosis |
| 721.5 | Kissing spine |  | *724.02* | | *Lumbar stenosis* |
| 721.9 | Spondylosis, unspecified site |  | 724.03 | | Lumbar stenosis with neurogenic claudication |
| 721.90 | Spondylosis without myelopathy |  | 724.09 | | Spinal stenosis, other |
| 722.2 | Displacement of disc without myelopathy |  |  | | |
| 722.5 | Degeneration, thoracic or lumbar disc |  | **Other Diagnoses** | | |
| 722.51 | Degeneration, thoracic or thoracolumbar disc |  | 733.1 | Pathologic fracture | |
| 722.52 | Degeneration of lumbar or Lumbosacral intervertebral disc |  | 733.10 | Pathologic fracture, unspecified site | |
| 722.6 | Degeneration of intervertebral disc, site unspecified |  | 733.13 | Pathologic fracture of vertebrae | |
| *724.2* | *Lumbago* |  | 733.95 | Stress fracture (not lower extremity) | |
| *724.5* | *Backache, unspecified* |  | 721.6 | Ankylosing vertebral hyperostosis | |
| 724.6 | Disorders of sacrum |  | 722.11 | Thoracic disc displacement | |
| 739.3 | Nonallopathic lesion, lumbar |  | 722.3 | Schmorl’s nodes | |
| 739.4 | Nonallopathic lesion, sacral |  | 722.93 | Other disc disorder, lumbar | |
| 846.0 | Sprains & strains, lumbosacral |  | 724.1 | Pain in thoracic spine | |
| 846.1 | Sprains & strains, sacroiliac ligament |  | 738.4- | Acquired spondylolisthesis | |
| 846.8 | Sprains & strains, other specified sites of sacroiliac region |  | 738.5 | Other acquired deformity of spine | |
| 847.2 | Sprains & strains, lumbar |  | 739.2 | Nonallopathic lesions, thoracic | |
| 847.3 | Sprains & strains, sacrum |  | 756.11 | Spondylolysis | |
| 847.9 | Sprains & strains, unspecified site of back |  | 756.12 | Spondylolisthesis | |
|  |  |  | 846.9 | Sprain, unspecified site, sacroiliac region | |
| **Back and Leg pain or herniated disc** | |  | 847.1 | Sprain thoracic region | |
| 722.1 | Displacement of thoracic or lumbar disc without myelopathy |  | 999.99 | Specialty code: non-specified indication | |
| 722.10 | Displacement lumbar disc without myelopathy |  |  |  | |
| 722.2 | Displacement of disc without myelopathy |  |  |  | |
| *724.3* | *Sciatica* |  |  |  | |
| *724.4* | *Thoracic or lumbosacral neuritis or radiculitis* |  |  |  | |
